# Australian general practitioner perceptions to sharing clinical data for secondary use: a mixed method approach

**DOI:** 10.1186/s12875-022-01759-y

**Published:** 2022-07-01

**Authors:** Richard J. Varhol, Sean Randall, James H. Boyd, Suzanne Robinson

**Affiliations:** 1 School of Population Health, Faculty of Health Sciences, Curtin University, Bentley, WA Australia; 2 Department of Public Health, School of Psychology and Public Health, College of Science, La Trobe University, Health & Engineering, Melbourne, Australia

**Keywords:** Primary care, Data collection, Secondary use, Electronic medical records, Governance, Stakeholders

## Abstract

**Objective:**

The potential for data collected in general practice to be linked and used to address health system challenges of maintaining quality care, accessibility and safety, including pandemic support, has led to an increased interest in public acceptability of data sharing, however practitioners have rarely been asked to share their opinions on the topic. This paper attempts to gain an understanding of general practitioner’s perceptions on sharing routinely collected data for the purposes of healthcare planning and research. It also compares findings with data sharing perceptions in an international context.

**Materials and methods:**

A mixed methods approach combining an initial online survey followed by face-to-face interviews (before and during COVID-19), designed to identify the barriers and facilitators to sharing data, were conducted on a cross sectional convenience sample of general practitioners across Western Australia (WA).

**Results:**

Eighty online surveys and ten face-to-face interviews with general practitioners were conducted from November 2020 – May 2021. Although respondents overwhelmingly identified the importance of population health research, their willingness to participate in data sharing programs was determined by a perception of trust associated with the organisation collecting and analysing shared data; a clearly defined purpose and process of collected data; including a governance structure providing confidence in the data sharing initiative simultaneously enabling a process of data sovereignty and autonomy.

**Discussion:**

Results indicate strong agreement around the importance of sharing patient’s medical data for population and health research and planning. Concerns pertaining to lack of trust, governance and secondary use of data continue to be a setback to data sharing with implications for primary care business models being raised.

**Conclusion:**

To further increase general practitioner’s confidence in sharing their clinical data, efforts should be directed towards implementing a robust data governance structure with an emphasis on transparency and representative stakeholder inclusion as well as identifying the role of government and government funded organisations, as well as building trust with the entities collecting and analysing the data.

**Supplementary Information:**

The online version contains supplementary material available at 10.1186/s12875-022-01759-y.

## Background

General practice is considered the cornerstone of healthcare and provides the foundations of a health system designed to protect and improve the health of the population [1, 2]. To assist in managing an increasingly complex patient demographic, general practices have taken advantage of advancements in compute power, storage capacity and the reduced costs of technology by incorporating digital technologies and electronic medical records (EMRs) into their clinical practice. Practitioners now have the capability to collect vast amounts of clinical data points on individual patients to assist them in the assessment and management of patients. Although patient data collected at point of care is primarily used for managing and treating an individual patient, when integrated across a network of health care providers [3, 4], these health systems can be used to improve clinical processes, care coordination, quality of care, time efficiencies [5, 6], patient safety [7], health outcomes [8] and more recently in pandemic support [9].

The untapped potential within these EMRs has garnered considerable interest from private and public sectors resulting in general practices increasingly being solicited by third parties to share patient data for a variety of purposes with varying degrees of participation. Several international jurisdictions have invested considerably in developing health system infrastructures for collecting and integrating general practice data, some of which include the recently launched GPDPR [10] as part of UK’s NHS Digital; Scotland’s SPIRE service [11]; Wales’ SAIL databank [12]; Denmark’s eSundhed [13]; Canada’s POPLAR network [14]; and New Zealand’s IDI [15]. Australia has undertaken similar implementation initiatives such as NPS MedicineInsight [16–18], LUMOS [19] and general practice data collected through the Primary Health Networks [18]. However, apart from New Zealand where all practice data is collected centrally, there has been limited success in attracting general practices to share their data beyond its use in direct care delivery.

Australian general practitioners are currently paid through the Australian government’s Commonwealth Medicare Benefits Schedule for the services they provide and are often in competition for patients [20] unlike their counterparts in the UK where patients are assigned a practice within their residential postcode. These Australian general practitioners operate as an independent business with narrow profit margins, requiring practice owners to have both financial and business acumen.

Although studies have been conducted to gauge patient [21–24] and researcher [25] sentiments on sharing clinical data, there is limited information on practitioner perception [26–30] to sharing patient information. Understanding practitioner perception is critical, as in most countries, practitioners are the custodians of their patient’s data and have ultimate decision as to its use. Using online surveys and in-person interviews, our research builds on previous findings [27] to enable a greater understanding of the associated barriers and facilitators for general practitioners to share their patient data for secondary use.

## Methods

### Study design

A mixed method approach was used to understand general practitioner’s perspectives on sharing health data utilising both an online survey and interviews. A cohort of Western Australian general practitioners was selected as part of ongoing engagement activities associated with the WA Primary Health Alliance. The online survey was conducted from November 2020 – May 2021 and consisted of 21 questions with 7 categories (refer to supplementary material). A total of 80 surveys were received; 16 of which were deemed to be incomplete resulting in a total of 64 which were used for analysis. Semi-structured in-depth interviews were used to supplement the survey, enabling general practitioner perceptions to be explored in greater detail by providing a broader context for interpretation of the survey results. Data collection for both surveys and interviews extended into the COVID-19 pandemic requiring careful planning to capture general practitioner responses, a cohort known for poor participation rates [31].

The survey and interviews consisted of six sections which explored a) previous participation in research; b) willingness to share practice data for secondary use including health research; c) type of data that could be shared; d) concerns related to sharing data for secondary use; e) data collection and storage and f) understanding of how the data was collected and stored.

These were subsequently synthesised into three broad areas of discussion of purpose and process of the secondary use request, trust in the organisation collecting and analysing the data, and governance associated with the program. Responses were aligned to assess the barriers and facilitators to data sharing, with the first two of all Likert scale responses combined for reporting purposes (e.g. very high trust and high trust have been combined in the results).

### Survey

To gather insights from a broad representation of general practitioners and to maximise the responses from the target audience, both on-line (REDCap electronic data capture tool hosted at Curtin University [32]) and printed versions of the survey questionnaire were produced. Survey questions were developed based on themes identified from previous Australian studies [17, 18, 27, 30], with additional input from 3 general practitioners. Focussing on trust and understanding, the questionnaire asked general practitioners the extent to which they would be comfortable with sharing their clinical data for secondary uses (i.e., beyond direct care delivery). Items were ranked on a 5-point Likert scale where 1 represented a strong positive association and 5 depicted an extreme distancing response. Prior to operationalising the survey, we piloted the questionnaire with six practitioners who were actively engaged with their local Primary Health Network (PHN), one of 31 federally funded organisations responsible for augmenting primary health care based on community need. Feedback from the pilot cohort returned an emphasis on relevance and clarity, resulting in modifications being made to the survey pertaining to improved terminology and the reframing of questions to avoid leading inquiries.

The survey consisted of 21 questions and was developed to work with the interviews through complementary themed questions around attitudes to data sharing.

Participants were recruited between February and November 2020 using Healthdirect – Australia’s online Healthmap portal containing a listing of 730 publicly listed general practices [33]. The link to the survey including the information pack consisting of a flyer advertising the study containing both the URL and a QR scanning code for smart devices, were sent to primary care organisations including education, training and workforce agencies who engage with general practitioners. In addition, general practices were directly approached, by phone, email and in-person, by the lead author to further promote participation.

### Interviews

To provide additional context to general practitioner perceptions about sharing patient data, a cohort of 10 practitioners from 100 engaged practices known to have previously participated in PHN led health care interventions (and independent of those involved in the survey development and pilot) were invited and subsequently consented to participate in an audio-recorded semi-structured interview. Eight of the interviews were conducted in person, one by phone and one using video conferencing technologies. The lead author conducted all interviews using an interview run sheet (supplementary material), to guide the open-ended discussion ensuring all points were covered.

Interviews ranged from between 30 – 45 min in length and were conducted in two batches, pre COVID-19 isolation activities (*n* = 5) and post (*n* = 5). Participants were contacted by phone and email, providing them with participant information sheets, consent forms and an invitation booking. Interviews were recorded using a digital recorder and transcribed manually within a week of the conversation. NVivo software (v1.5) [34] was used based on a themed template analysis approach [35] with a hierarchical coding template was developed on a subset of data which was refined and revised throughout the process. The codes were subsequently themed and applied by selecting illustrative quotes from participant. Superfluous text was removed and replaced with ellipses […].

### Ethics

This research project was approved by the Curtin University Human Research and Ethics Committee (HREC) (approval number: HRE2019-0619–02).

## Results

A total of 80 surveys were submitted of which 64 were completed. Of the surveys that were completed, 17 (27%) were collected prior to the start of the COIVID-19 pandemic. Sixty-seven percent of general practitioners that completed a survey were over the age of 40 years and of these, 38% identified as actively practicing medicine between 11–30 years. Characteristics of the participants are available in Table 1. Of the 10 practitioners who were interviewed, eight identified as either being part owner or sole proprietor of their practices.

**Table 1:** Interview and survey participant characteristics

Characteristic	Surveys n (%) Total = 64	Interview n (%) Total = 10
Age Group (Years)		
< 40	21 (32.8%)	1 (10.0%)
40–55	19 (29.7%)	4 (40.0%)
> 55	24 (37.5%)	5 (50.0%)
Years Practicing		
0–10	31 (48.4%)	4 (40.0%)
11–30	24 (37.5%)	5 (50.0%)
> 31	9 (14.1%)	1 (10.0%)
Practice Size		
Solo	1 (1.6%)	-
2–5 general practitioners	8 (12.5%)	1 (10.0%)
6 + general practitioners	55 (85.9%)	9 (90.0%)
Practice Owners	NA	8 (80.0%)
Location (PHN)		
Perth North [Urban]	28 (43.8%)	4 (40.0%)
Perth South [Urban]	19 (29.7%)	4 (40.0%)
Country WA [Rural and Remote]^a^	3 (4.7%)	2 (20.0%)
Not Disclosed	14 (21.9%)	-

*NA* Not Asked, *PHN* Primary Health Network,

^a^As defined by the Modified Mixed Monash model [36]

The survey and interview responses were collected across six themed sections (Table 2) to gather information on the practitioner’s experience and willingness to participate in research, their understanding of the data sharing requirements and the concerns that they had. Aggregation of Likert scores occurred for survey questions where granularity resulted in ambiguity or indifference.

**Table 2:** Summarised themes obtained from interviews

Main Themes	Sub Themes
Previous participation in research	• Lack of knowledge related to requirements • Imposed health system regulations • Impact to business ○ Time ○ Consent ○ Liability
Willingness	• Acknowledgment of data already being shared • Consequences due to wrong conclusions: ○ Litigation ○ Misinterpretation ○ Loss of business
Types of data	• Agreeable to share de-identified aggregate data • Non-threatening • Caution related to business impacts
Data sharing concerns	• Organisational ○ Trust in collecting and storing data ○ Financial backing ○ Reputational risk
Data collection and storage	• Larger, government agencies garner more trust • Universities respected for ethics and research protocols
Understanding	• Concerns related to lack of transparency, autonomy and sovereignty • Confidence related to privacy legislation obligations

More detail and illustrative quotes are included below

### Previous participation in research

A majority of those surveyed (*n* = 54, 84%) had participated in research on more than one occasion, with 95% of practitioners stating research to be either important (*n* = 17, 26%) or very important (*n* = 44,69%) to Australia’s future. Given the opportunity of participating in future research projects, 67% of participants considered it very likely (*n* = 23,36%) or likely (*n* = 20, 31%) of being involved, with 30% (*n* = 19) feeling uncertain.

When specifically asked about sharing data for research purposes the majority of practitioners interviewed (*n* = 6, 60%) indicated that a lack of knowledge about research requirements was a barrier, with one practitioner stating:

“It’s not that we’re not interested or don’t want to be involved in research, it’s just that we’ve never done it and don’t know where to start. ”

Another highlighted regulation imposed within the health system as being the main impediment to sharing data for broader consideration:

“The system is more regulated than it used to be, to the point that it is ridiculous how careful we have to be. I suppose it’s this that prevents us from sharing data outright. Even though the technology makes it easier to share data the bureaucracy and the amount of associated regulation is slowing things down.”

When asked what factors would prohibit participation in research projects, respondents identified time required to thoroughly understanding the purpose of request and obtaining consent as the main challenges.

Practice owners (*n* = 8, 80%) who had been asked to participate in previous data sharing projects were expected to expertly review each request and familiarise themselves with all aspects of the project. This includes understanding and agreeing to the purpose of the data request; assessment of clinical and legal risks to both the patient and the practice; impact to the business associated with the time required to collect, prepare and securely transfer the data; ascertain the liability, accountability and extent of coverage specified in the related data sharing agreement; as well as placing trust in the organisation requesting the data.

“…most of the time we don’t have a good understanding of what the data is being collected for and the data has the potential to be used out of context and even though it may be telling you something it may not be representative of what is actually happening in the practice. To get to that level of understanding I would need to put in dedicated amount of time to match my level of understanding to what is being asked for. It’s a fear of not knowing. Sometimes you just have to blindly trust what they are telling you what the data will be used for.”

Additionally, these concerns led some practitioners to question their role in primary care, referring to the administrative overhead required for understanding and approving individual projects or understanding research and information governance.

“….from a collection stand point … is that going to take away valuable time I have with the patient? Am I now going to be a collector of data rather than a listener of a story to a patient? Will I be taken away from my primary purpose for which I am there?”

Patient recruitment and obtaining consent requires an investment of valuable time outside the therapeutic relationship between practitioners and their patients.

“Consent is cumbersome and time consuming; however, there is a lot of value in that because you’re building a relationship with the patient. You’re building an understanding of the project and the natural progression from that is whether they’re happy and if all their questions have been answered before they consent.”

Depending on the data request purpose, practices may be required to obtain consent from both patients and the practice general practitioners, adding an additional level of complexity and time.

“My reluctance is going to everybody [in the practice] and getting permission. There is a perception that the data belongs to them [general practitioners], and that’s a bit of a grey area. Because the data doesn’t belong to them, it belongs to the practice and that could confuse things.”

### Willingness

Willingness to share data for secondary use was ascertained by the practitioner’s perceived risk to their patient, other health providers involved in the patient’s care, or their general practice. The majority of respondents felt there was very low or low risk to the patient (*n* = 52, 81%), other health providers (*n* = 47, 73%) or to the general practice (*n* = 43, 67%), if de-identified patient information were to be used for research purposes Interviewed practitioners expanded on their willingness to share their patients’ data for secondary use to include data sharing across the health care system which is an established part of their clinical workflow.

“Look, we already share quite a bit of patient information – as part of their care plans with other GPs including path labs and radiology clinics…so I’m comfortable with this from that approach.”

“To be honest, whether I give you the data for research or to the federal government, I’d have the same concerns … what will you be doing with data, what are you going to do with the data, is my data safe, who’s benefitting and how … and can I trust you?”

When asked to identify the potential consequences to sharing data for secondary use, surveyed practitioners, identified data breaches (*n* = 50, 78%), misinterpretation of data leading to wrong conclusions for both the practitioner (*n* = 37, 58%) and the practice (*n* = 33, 52%) as the top three consequences. Information used for outcome payments (*n* = 29, 45%), litigation (*n* = 25 39%) and potential loss of patients (*N* = 14, 22%) featured less as concerns. Eight survey respondents offered additional consequences which largely reflected their concerns with sharing patient data. These included a lack of clarity with regards to consent (*n* = 2, 2.8%), extra time required in an already busy practice (*n* = 2, 2.8%), data entry errors resulting in unreliable, non-uniform data leading to wrong conclusions (*n* = 3, 4%), and a threat to funding and revenue streams for general practice (*N* = 1, 1%).

### Types of data

Depending on the type of data being requested, surveyed practitioners were comfortable (combination of ‘very’ and ‘somewhat comfortable’) in sharing demographic information (*n* = 59, 92%), medical information (*n* = 55, 86%), location information (as postcode) (*n* = 50, 78%), family history (*n* = 44, 69%), hospital information and community care information (*n* = 36, 56%). However, personally identifiable information (such as name, birthdate, phone number) was recognised as being sensitive, with high (*n* = 48, 75%) levels of discomfort and concern around secondary research use.

Sharing of non-threatening aggregate data was found to be more acceptable for most of the interviewed practitioners, with many participating in the federal government’s Practice Incentives Program for Quality Improvement (PIP QI), a financially incentivised program supporting quality improvement activities [37, 38]. However, some concerns still existed where it was perceived that the data would expose a practice’s business intelligence:

“Even if you’re sharing high level aggregate data like number of active patients or number of patients with a chronic disease for example, you are potentially encroaching on the practices business intelligence and may give insights to our competitive edge and could be used in inappropriate ways. After all we’re all in the market competing for the same patients. At the end of day, we are all private business and are operating competitively.”

“I’m happy to share data that’s aggregated and nothing else. We already do this to claim the PIP payment. We reviewed this within the practice and found that it was of low risk to both our patients and the practice … for the moment … because we don’t know what else will it be used for.”

### Data sharing concerns

**Fig. 1:**
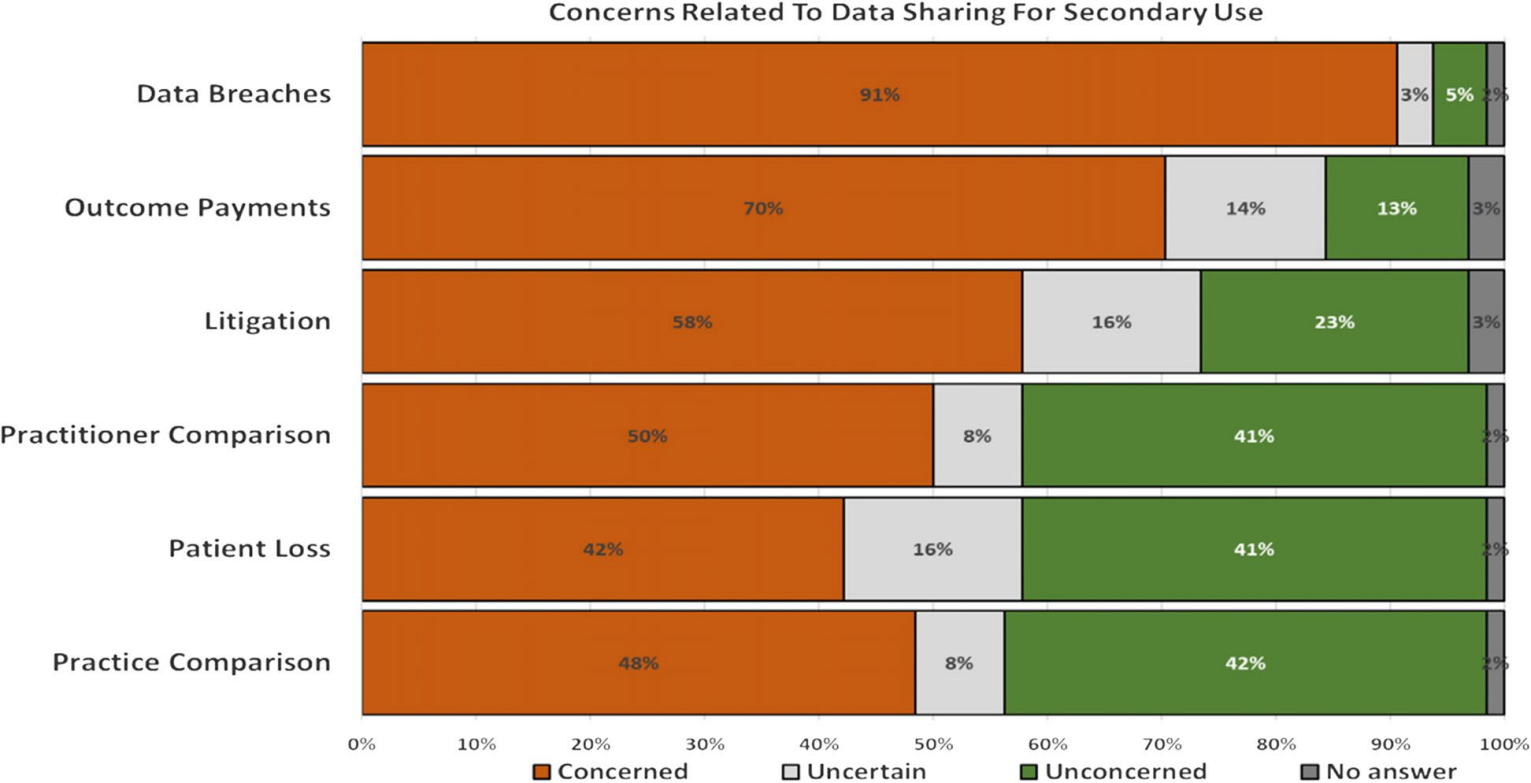
Concerns related to data sharing for secondary use

When asked what would prevent practitioners from sharing their data with the Commonwealth agencies, data breaches (*n* = 58, 91%), data being used to tie general practice funding to outcome payments (*n* = 45, 70%) and litigation (*n* = 37, 58%) ranked as the three main concerns (Fig. 1). Interviews confirmed that trust in an organisation’s ability to collect, store and be responsible for the shared data in a secure manner as well as financial backing, can influence a practitioner’s willingness to share their data for research and secondary use.

“I would have trust in organisations that have the layers of governance that say the government has. It’s not just the data breaching element, it also includes what level of authority that data carries and also the amount of support, money and finances are behind them to provide that level or protection and what level of risk to reputation can mean for that organisation”

“It’s not that we have potential treasure in the data we’re providing that we have to protect – the treasure is in the relationship we have with our patients…. Trust comes from actions…. It takes five years to build it and only two minutes to destroy it.”

“If there is a data breach … is the practice indemnified? If the patient decides to sue, whom could they go after?”

Upon sharing their data with a third-party, interviewed participants noted considerable concerns relating to lack of transparency with how the data would be managed and used, including a loss of control over what happens to the data. Relinquishing control of the data places some practices in a vulnerable position with many becoming both cautious and sceptical as to how it will be used.

“At the end of the day there is a bit of a trust issue with the requestor. This is dependent of what they do with the data. Why are they paying for the data? How will we know that the data is only being used for the intended purpose and not for something else? The person who is asking for the data is usually the same person that punishes you.”

“Nobody knows what the government will do with the data making us [general practitioners] feel just as uneasy sharing our data as with a third-party organisation without proper data sharing agreement in place. Improving the clarity of how the data will be used would help build confidence….The rules are complicated and very volatile.”

“Basically, I want to know what the intended purposes are. I mean what are they going to do with our data? How will the data that I give to the third party be used? I don’t want it to be manipulated, misconstrued or misinterpreted. The reason for that is that I don't want to lose control. Not control is one thing, but not having a say in how the data gets used for secondary use is another.”

### Data collection and storage

Surveyed practitioners were asked about the perceived trustworthiness of Australian organisations collecting various forms of health data. Government agencies were generally perceived as having higher levels of trust. The Royal Australian College of General Practice (RACGP) (*n* = 30 47%), the largest rganization representing general practitioners, was also considered to be a trusted entity for data collection and storage. Primary Health Networks (*n* = 31, 48%), Australian Digital Health Agency (*n* = 27, 42%) and Universities (*n* = 26, 41%) were all perceived as having a moderate level of trust; while corporate organisations (*n* = 58, 91%) such as commercial tracking companies, software companies and extraction vendors were perceived to have the lowest trust.

Sharing data with a university provided reassurances that data would only be used for a specific purpose with no potential of over-reaching or scope creep. This is in part attributed to the ethical guidelines and principles universities are required to follow when working with health-related data. As one practitioner noted:

“I’m reassured with university research as I know if the project has ethics approval, it can only be used for a specific purpose and nothing else. I’d be happy for the university to have control of the data from an academic perspective because universities tend to have a lot of respect across the clinical and research community…”

### Understanding

When practitioners were asked about their level of understanding of how shared data would be managed across different organisations, most of the respondents had little (combining familiar and somewhat familiar responses) to no familiarity with the processes, with an average of 32 and 66 percent respectively (even though many had a medium to high level perception of trust).

Interview participants noted considerable concerns relating to lack of transparency, autonomy and sovereignty around shared data, including a loss of control over what happens to the data. Relinquishing control of the data places some practices in a vulnerable position with many of them being both cautious and sceptical.

“Look, we’re a business - just like all the other practices – and we need to take advantage of whatever funding opportunities come our way and you can argue that PIP is one of those opportunities. We wouldn’t share otherwise. We upload our data to the PHN who in turn do who knows what with it… I expect that eventually the data will be used in some way to get more out of us. Without context the data could actually be misrepresenting us and that’s a concern. It would be nice to have more control as we currently don’t really know how are data is being used and for what purpose.”

“I’d like more control over my data…. being able to opt in or out. Ensuring that the data would not be used for punitive purposes…it’s about not knowing what they will be doing with the data in the future.”

Overall, 89% of practitioners were considered to be confident (combination of extremely, fairly and somewhat confident) in understanding Australia’s privacy legislation. Seventy-five percent of respondents identified having a reliance on the RACGP to provide guidance (‘fairly confident’ [*n* = 29, 45%] and ‘somewhat confident’ [*n* = 18, 28%]). A small group (*n* = 10, 16%) identified as being extremely confident by having read the legislation and had a complete understanding of their obligations.

“I understand the ethical considerations of the Privacy Act. I’ve recently had to revisit this for the eHealth application [My Health Record] that was rolled out recently. Where I have to call my patient in and explain to them and give them an explanation statement. I have to make sure they fully understand why I'm sharing their data with this organisation.”

## Discussion

Our paper identified Western Australian general practitioners as having an appreciation of the importance of primary care data for secondary use, this is consistent with earlier Australian studies [27, 30]. However, there was an apprehension with sharing their data due to potential data breaches, misinterpretation of the data collected, impact on business, lack of knowledge or research requirements and trust in and the financial backing of the organisation collecting and analysing their data. These observations reflect previous studies [26, 27, 39].

Depending on the purpose, practitioners had misgivings to what and how long their data was used for, including the potential for misrepresentation given the potential lack of understanding around the context associated with the collected data. Practitioners who previously participated in research projects were more likely to consider the request because of the associated ethics approval which stipulates the scope and limitations of what the data can be used for, including the consideration of participant consent if required. In lieu of a national privacy policy, health providers must be extra vigilant in their custodianship role by assessing the potential risk to their patients [40]. While ethics approvals are essential for research studies they provide a level of surety for both the patient and general practitioner, ensuring that the collected data will be used as stipulated, it can be an impediment to a rapidly evolving data landscape [41]. Related concerns have not been reported in New Zealand where a national privacy policy has been developed and implemented [6, 42, 43]. An additional facilitator to the New Zealand context is the availability of unique health identifier for each citizen, which when used in the auspices of the privacy policy, can be deterministically linked to the Integrated Data Infrastructure [15] without any additional personal information, providing more information about healthcare service availability and utilisation.

The lack of a clearly defined, nationally approved, data sharing framework [27, 44] has further detracted Australian general practice from investing time to familiarise itself with each data request to confidently share their patient data for secondary use. Hesitation in participating in data sharing activities related to a general lack of understanding that went beyond how the collecting organisation was managing, processing and analysing their data asset. This included confusion associated with data ownership within the practice and whether each practitioner was required to provide approval to participate. If general practice lacks clearly defined data ownership policies [45] or if the individual practitioners are not aware of them; difficult discussions about who owns the data may play an additional barrier to sharing data. An acknowledgment of frustration in their inadequate understanding of what was being asked of them as part of the data request, originated from the amount of time was necessary to become familiar with the project.

General practices in Australia are independent businesses in competition with one another. Unlike those in New Zealand and the UK, Australian general practice required the principal practitioner(s) to be experienced in all aspects of running a business in addition to maintaining clinical accreditation and providing care; all within a limited time frame and tight budgetary constraints [46]. Dedicating time to be informed about the project, collect, review and validate the data and potentially collect consent, provides challenges for general practice simultaneously questioning the practitioner’s primary role as health provider, becoming more of a health care data administrator.

In Australia, over 72% percent of general practitioners receive a financial reimbursement from the federal government for voluntarily providing their de-identified patient data as part of PIP QI program [37]. Through the quarterly submission process orchestrated by the PHNs, practices are further supported through the provision of data extraction software and practice specific data quality reports.

The reports provided by the PHN allow general practices to review and improve their data quality, with some practitioners hesitantly anticipating these payments to ultimately be associated to outcomes as was implemented in the UK with the Quality Outcomes Framework (QOF). Although the benefits of the QOF are yet to be determined [47, 48], the adoption of a pay for performance program was unpopular with many practitioners at the time, which could be a contributing factor to the low uptake of voluntary secondary use data programs. A similar observation was shared amongst several Australian practitioners that participated in a national study related to the PIP QI implementation [49] generating division and confusion across general practice in participation of the program.

The potential implementation of an outcome-based funding model together with the uncertainty as to how the collected general practice data will be used, has made Australian practitioners weary and concerned their data will be used against them and disrupt the current business model. Sub-themes of autonomy and data sovereignty were identified throughout the interviews with practitioners wanting to be able to opt-out of data sharing program in light of the undefined scope of what the data will be used for. Practitioners may benefit financially from data sharing for payment incentives; however, some have questioned the administrative time required to complete the claims which would be better spent with their patients. A similar sentiment was seen in the UK with physicians being over burdened with increased administrative workloads resulting in increased work stress [50].

Another area of concern for general practice is with the current funding model of claiming for provided medical services and prescribing medication. In Australia, general practitioners are more prone to being audited than any other healthcare professional, resulting in revenue loss and reputational damage should a claim of inappropriate service provision or prescribing be made. Although a hyper vigilant approach to health care provision may be conducive to optimal care, it is yet another barrier to overcome, as general practitioners feel scrutinized at multiple levels.

### Removing barriers

Practitioners’ hesitation to participate in secondary use data projects is substantiated through numerous unsolicited data requests from across the health system. Absence of a coordinated system devoid of standards has developed a culture of trepidation and resistance. These findings are supported by previous Australian studies [3, 18, 27, 30] and highlight the need for transparency, establishment of independent data governance committees, defined scope pertaining to data utlilisation and development of a broad set of best-practice principles to sharing health data [18]. Similar approaches have proven to be effective in jurisdictions such as the UK [11, 51, 52], Denmark [13], Canada [53, 54] and New Zealand [40].

Development of these guidelines is further supported by the creation of the National Primary Health Care Data Collection. This initiative, led by the Australian Institute of Health and Welfare [55] will consolidate existing primary care data assets, governance processes and standards in an integrated environment, with the aim of addressing health system gaps through standardised data collection and normalised reporting. Unlike some large scale, national health data integration projects challenged by complexity and enormity of scale [56–58], the National Data Collection can utilise the recently implemented Primary Health Insights platform, for the secure storage and analytics of primary health care data and utilised by 27 of the 31 PHNs to store their primary care data.

In addition to the establishment of a transparent authoritative governance structure, the collection, analysis and storage of the data facilitated by a trusted federal entity further reduces the barrier for data sharing. Trust in this context is associated with national prominence and reassurance that appropriate compensation (financial and legal) could be obtained should it be required. This is seen in other countries where centralised data collections are managed and funded in part by federal bodies. With the recent implementation of national storage and analytics platform [59], the Australian government has funded the PHNs to manage and continue to develop their relationships with general practice to share their data for quality improvement activities.

Coinciding with the national data platform implementation, the Australian government restructured the practice incentive payment program by compensating general practice financially for sharing their data through their PHN who in turn provide reports and improvement activities, assisting practices to achieve quality improvement and patient outcomes.

### Strength and limitations

This study utilised a mixed-methods approach, providing in-depth information from the small number of interviewees complemented by the larger number of survey respondents. Considerable effort was made to promote the survey and recruit across the practitioner network, resulting in 80 survey submissions. This was considered a successful result given general practitioner recruitment for research is acknowledged as a major challenge for researchers [60, 61] which for this study was further compounded by the COVID-19 pandemic which restricted survey promotion. General practitioners who completed the survey may be more likely to be involved with research and may have different views to the barriers compared to those who did not complete the survey; however, those general practitioners more involved in research may also have a greater understanding of the current barriers. Additionally, this paper looked only at Western Australian general practitioners; it cannot be guaranteed that the experience of these practitioners is reflective of those in other Australian states. Although general practitioners operate within the same funding arrangements; the data programs they participate in may differ between states, resulting in different perceptions to data sharing.

## Conclusions

Previous authors have identified several benefits related to sharing and integrating routinely collected patient data from general practice [5–9] These advantages are contrasted by barriers including trust in what the data will be used for, lack of knowledge of the process, no financial incentive, concerns related to litigation, and data breaches which restrict the efficient use of these data. These barriers can in part be addressed through the establishment of a local clinical governance council, as well as a federal entity to facilitate the collection, storage and analysis of data. Understanding these barriers will have a fundamental contribution to increasing general practitioner participation in secondary-use projects and programs to facilitate these data resources to their fullest potential.

## Supplementary Information

**Additional file 1:** (ZIP 664 kb)

## Data Availability

The datasets generated and/or analysed during the current study are not publicly available due [due to ethics committee requirements and the need for further participants’ consent] but are available from the corresponding author (after obtaining further ethics approval) on reasonable request.

## Abbreviations

COVID-19: Corona Virus Disease 2019
EMR: Electronic Medical Records
HREC: Human Research and Ethics Committee
PHN: Primary Health Network
PIP QI: Practice Incentive Program for Quality Improvement
RACGP: Royal Australian College of General Practitioners
UK: United Kingdom
URL: Universal Resource Locator
QOF: Quality Outcomes Framework
QR code: Quick Response code
WA: Western Australia
