# Supplementary material for: Australian general practitioner perceptions to sharing clinical data for secondary use: a mixed method approach
Source: BMC Prim Care. 2022 Jul 1;23:167. doi: 10.1186/s12875-022-01759-y (PMC9247967; doi:10.1186/s12875-022-01759-y)
Supplement: Supplementary file 1 — Additional file 1: (ZIP 664 kb) [file 12875_2022_1759_MOESM1_ESM.zip › INFORMATION_SHEET-Facilitators_and_Barriers_To_Sharing_Health_Data-20200113_v0.5.pdf]

PARTICIPANT INFORMATION SHEET

**HREC Project Number:**	HRE2019-0619-02
**Project Title:**	Exploring the barriers and facilitators to sharing health data
**Chief Investigator:**	Richard Varhol, Project Lead, Curtin University
**Version Number:**	0.5
**Version Date:**	12/02/2020

This Participant Information Sheet tells you about the project. It explains what will be involved and helps you to decide if you would like to take part. Please read this information carefully. If you have any questions about anything you don't understand or want to know more about the project, please contact the Chief Investigator (Richard Varhol) at rvarhol@curtin.edu.au. By agreeing to participate in the study you are telling us that you:

- Have read this Participant Information Sheet
- Understand what you have read
- Consent to take part in the project
- Consent to the data collection that is described

If you have any queries, would like additional information or wish to speak with the research team please email or call: Richard Varhol at rvarhol@curtin.edu.au or 08-9266-3495

What is the Project About?

This research is one of the first studies to lay the foundations and explore the possibilities of integrating primary and secondary care data across the continuum of care in Australia. This study will investigate the possibilities for data integration across the continuum of care by identifying relevant information necessary to inform clinical management and population health planning. In consultation with health providers, this research is aimed at developing and evolving the appropriate clinical and policy planning questions in light of the data that is currently collected in the practice and available through other areas in the health systems.

This research will also support the recommendations from the Productivity Commission's report around prototyping and developing accredited State based entities for data linkage. The results from this research will contribute to an evidence base, which is currently limited in supply; while at the same time adding translational value to both general practice and the broader health system, by providing unique insights into Potentially Preventable Hospitalisations (PPHs) which is a National priority for all Primary Health

Networks (PHNs) and will help address some of the recommendations of WA Department of Health's Sustainable Health Review.

Who is doing the Research?

The project is being conducted by Richard Varhol, who is enrolled in the PhD program at the Curtin University. There will be no additional cost to participate and you will not be paid for participating in this project.

Why am I being asked to take part and what will I have to do?

You have been asked to take part in this project because as a General Practitioner, you may have been approached by various organisations to share your practice data, and we would value your expertise in the area of data extraction and data sharing. If you agree to participate in the research, you will be asked to complete an online questionnaire which will take between 5-10 minutes of your time.

Optional consent

You will also be asked to indicate consent at the beginning of the questionnaire.

Are there any benefits' to being in the project?

Apart from the knowledge that your answers to the questions are contributing to an important topic and ongoing debate of data sharing across primary care, which may be included as part of broader body of work; there are no immediate benefits. Being part of this research will however provide you with the opportunity to have your say on this topical subject. The results will offer support to health care providers, policy makers and researchers, that are interested in utilising routinely collected data to improve innovations around clinical evaluation and business planning. It will also enable the researchers to better identify the health care gaps and to collaboratively plan services and support functions more appropriately.

Are there any risks, side-effects, discomforts or inconveniences from being in the project?

There are minimal foreseeable risks involved from being in this research project. Apart from giving up your time, we do not anticipate that there will be any risks or inconveniences associated with taking part in this study. We have been careful to make sure that the questions in the survey do not cause you any distress. But, if you feel anxious about any of the questions you do not need to answer them.

Who will have access to my information?

The information collected in this project will be non-identifiable. This means that we will not be collecting any data that can identify you. All of the surveys will be coded with a unique identifier which will reside in a secure IS027001 environment hosted at Curtin University. Any information we collect will be treated as confidential.

Any information collected in this study will not be published in any manner that could identify you as an individual, during or after the conclusion of the project. The results of this research may be presented at conferences or published in professional journals. You will not be identified in any results that are published or presented, with only aggregated data being utilised.

Any hard copy information that we collect for the purposes of this project will be stored in secure locked facilities accessible only by the project investigators at Curtin University. Electronic information will be stored in the highly secure ISO-certified Centre for Data Linkage at Curtin University. All information collected for this project will be retained for seven years following completion. Hard copy information will be shredded and destroyed, and electronic data will be permanently deleted after this seven-year period.

Will you tell me the results of the research?

If you are interested in obtaining a summary of the results please contact the researchers after 12 months from the completion of the survey. Results will not be individual but based on all the information we collect and review as part of the project. We also plan to make non-identifiable aggregate results available in a scientific journal publication, so the learnings from our work can be shared with others seeking to get an understanding of the facilitators and barriers to data sharing across primary care.

Do I have to take part in the project?

Taking part in this project is voluntary. It is your choice to take part or not. You do not have to agree if you do not want to. If you decide to take part and then change your mind, that is okay, you can withdraw from the project. If you choose not to take part or start and then stop, it will not affect your relationship with the University, staff or colleagues. You do not have to give us a reason.

What happens next and who can I contact about the project?

If you would like to discuss the research further, or have specific enquires related to this study please contact:

Name: Richard Varhol **Phone:** 08-9266-3495 **Email:** rvarhol@curtin.edu.au

If you decide to take part, please select the box in the survey which indicates that you are agreeing to participate in the study. By agreeing to participate in this study you are telling us that you understand

what you have read. Please take your time and send any questions to the Chief Investigator before you decide what to do. You can print a copy of this Participant Information Sheet for your reference.

Curtin University Human Research Ethics Committee (HREC) has approved this study (HREC number HRE2016-0619-02). Should you wish to discuss the study with someone not directly involved, in particular, any matters concerning the conduct of the study or your rights as a participant, or you wish to make a confidential complaint, you may contact the Ethics Officer on (08) 9266 9223 or the Manager, Research Integrity on (08) 9266 7093 or email hrec@curtin.edu.au.
