# Supplementary material for: Australian general practitioner perceptions to sharing clinical data for secondary use: a mixed method approach
Source: BMC Prim Care. 2022 Jul 1;23:167. doi: 10.1186/s12875-022-01759-y (PMC9247967; doi:10.1186/s12875-022-01759-y)
Supplement: Supplementary file 1 — Additional file 1: (ZIP 664 kb) [file 12875_2022_1759_MOESM1_ESM.zip › Interview_Guide-Facilitators_and Barriers-Openended_Questions-Objective-1-Ver_1.0.docx]

**Important: Obtain consent (PICF_Version_1.docs) prior to continuing with the interview**

**Facilitators and Barriers to Data Sharing:**

| **Theme** | |
| --- | --- |
| **Understanding what is meant by data sharing** | |
| **Questions** | **Probe** |
| - What is your understanding of what data sharing is? - Can you give me some examples of how clinical / patient data is shared elsewhere? | - Wanting to gauge the level of understanding of data sharing |
| **Current data sharing status** | |
| - Do you currently share any practice and / or patient data? | - Some practices may already be sharing data, but not be aware. Ask if they are using:   - My Health Record   - Secure Messaging |
| **Willingness of sharing data** | |
| - How comfortable are you with sharing you clinical / patient data? - What data would you be comfortable sharing? | - How often would they be willing share? Quarterly, monthly, real-time? - How generalisable is the data you collect for research? - Do you collaborate with other institutions (i.e. PHNs, Universities, State Department, Clinical trials) |
| **What type of data** | |
| - Business related data (i.e. number and type of health providers rostered per day, patients not attending, etc.) - Deidentified patient and associated clinical data. - Identifiable patient data | - Supply versus demand - Patient treatment plans, date of diagnosis, health providers associated with patient management - What types of data do you collect? (i.e. progression of treatment, patient follow-up) - How do you collect it? - How do you deal with missing data? |
| **Who would you be willing to share data with?** | |
| - Within the practice - Between practices - With the State Health Department - With the Commonwealth - With other health providers (i.e. allied health) - Independent third parties (i.e. universities) | - Identify the types of data and with whom the respondent would be willing to share |
| **For what purpose would you be willing to share this data?** | |
| - Business Intelligence - Population health planning - Research - Practice Incentive Payment (PIP) - Improved patient management (trending, analytics, etc) |  |
| **Privacy and Security** | |
| - If you where to share your data, how would you like to do this? | - Cloud infastructure |
| **Data Governance** | |
| - If you were to share your patient / practice data, what data governance structure would you like to see? | - Ask who would be most trusted |
| **Patient Consent** | |
| - If you were share patient data what level of consent do you think would be appropriate? - And for what type of sharing | - Informed patient consent - Waiver of consent - Implied consent |
