# Supplementary material for: Australian general practitioner perceptions to sharing clinical data for secondary use: a mixed method approach
Source: BMC Prim Care. 2022 Jul 1;23:167. doi: 10.1186/s12875-022-01759-y (PMC9247967; doi:10.1186/s12875-022-01759-y)
Supplement: Supplementary file 1 — Additional file 1: (ZIP 664 kb) [file 12875_2022_1759_MOESM1_ESM.zip › SURVEY-Facilitators_and_Barriers_To_Sharing_Health_Data-HardCopy-20200202_v0.3.pdf]

Curtin University

Barriers & Facilitators to Sharing Health & Medical Data

You have been invited to complete a survey about your opinion of the use of health and medical data for research and long-term management and storage. This survey should take approximately 10 minutes to complete. The information collected in this study will help researchers and health professionals better understand general practice attitudes towards sharing and integrating data for health research and data linkage.

Knowing this information can enable researchers and health professionals to improve data collection and understand any concerns associated with data sharing and / or data linkage.

Please read the Participant Information Sheet before starting the survey.

If you have any questions, please contact Richard Varhol at: rvarhol@curtin.edu.au

By choosing to take part in this survey, you are consenting to participate which indicates that you:

- Have read the Participant Information Sheet;
- Understand the purposes, study objectives and risk of the project;
- Freely agree to participate in this study as described and understand that you are free to withdraw at any time during the survey;
- Understand that you can download a copy of the participant information sheet via the above link
- **Thank you for your time and feedback!**

A Note on Privacy:

This survey is anonymous. The recording of your survey responses does not contain any identifying information about you.

Curtin University Human Research Ethics Committee (HREC) has approved this study (HREC number HRE2019-0619-02). Should you wish to discuss the study with someone not directly involved, in particular, any matters concerning the conduct of the study or your rights as a participant, or you wish to make a confidential complaint, you may contact the Ethics Officer on (08) 9266 9223 or the Manager, Research Integrity on (08) 9266 7093 or email hrec@curtin.edu.au

Alternatively, scan or enter the URL into your browser to complete the survey online **http://j.mp/2T0BMJk**

☐ I agree to participate in the research study. I understand the purpose and nature of this study and I am participating voluntarily. I understand that I can withdraw from the study at any time, without any penalty or consequences.

Section A: Background Information

1. **What is your age range?**

   ☐ < 40 years old ☐ 40-55 years old

   ☐ 55+ years old ☐ Prefer not to say

2. **How many years have you been practicing as a GP in primary care?**

   ☐ 0-10 years ☐ 11-30 years ☐ 31+ years

3. **What is the size of the practice you work in** (i.e. nurses, nurse practitioners, other GPs)**?**

   ☐ Solo ☐ 2-5 practitioners ☐ 6+ practitioners

4. **In what Primary Health Network (PHN) is your practice located?**

   ☐ Perth North PHN ☐ Perth South PHN ☐ Country WA PHN ☐ I don't know

5. **Are you familiar with the Quality Outcomes Framework (QOF) which was implemented by the NHS in England, Wales, Scotland and Northern Ireland, as a system for performance management and payment of general practitioners?**

   ☐ Yes ☐ No

6. **Are you supportive, <u>in principle</u>, of the Quality Outcomes Framework?**

   ☐ Yes ☐ No

7. **Are you supportive, <u>in theory</u>, of the Quality Outcomes Framework?**

   ☐ Yes ☐ No

8. **Have you ever practiced in a jurisdiction where the Quality Outcomes Framework (QOF) was implemented (i.e. England, Wales, Scotland or Northern Ireland)?**

   ☐ Yes ☐ No

Curtin University

Section B: Public Health Research

The RACGP defines the Secondary use of data, as General Practice data used for purposes other than which it was originally collected.

This includes activities such as research, quality and safety measurement, provider certification or accreditation and marketing.

Potential public health gains from secondary use of de-identified patient data include clinical research outcomes, better informed epidemiological research, and improved service planning and quality assurance.

9. Not including this survey, have you ever participated in a research project?

☐ Yes, more than once ☐ Yes, on a single occasion ☐ No

10. In your opinion, how important is population health research to Australia's future?

☐ Very Important ☐ Important ☐ Moderately Important
☐ Slightly Important ☐ Not Important ☐ No answer

11. How likely are you to participate in a health or public health research project in the future?

☐ Very likely ☐ Likely ☐ Unsure
☐ Less likely ☐ Not likely ☐ No answer

Thank you for your time and feedback

Section C: Data Sharing & Secondary Use of General Practice Data For Research Purposes

Researchers can use different information to learn about people and their health.

For example, over the last few decades there has been a rise in the number of young people being diagnosed with asthma. Researchers have put together a database of information about the environment. They now want to associate the environmental observations with medical observations with people diagnosed with asthma. To do this, researchers look at asthma patients from General Practice records in a de-identified / anonymous way and associate them with environmental data in specific regions. By associating these two data sources (through a process called Data Linkage) researchers can identify potential trends in the data such as an increase in the number patients associated with an increased volume of traffic, which can then be used to inform policy makers and health professionals to improve patient outcomes.

The following question is focused on recording your thoughts and views related to the risk level of sharing data for research purposes for: Patients, Health Providers and General Practice.

12. How much risk do you think is associated with participating in research that involves patient de-identified information?

	Very low risk	Low risk	Moderate risk	High risk	Very high risk	No answer
Patients	☐	☐	☐	☐	☐	☐
Health Providers	☐	☐	☐	☐	☐	☐
General Practice	☐	☐	☐	☐	☐	☐

13. What do you think are the potential consequences? (Select all that apply)

☐ Wrong conclusions drawn in comparing practices

☐ Wrong conclusions drawn in comparing other practitioners

☐ Potential loss of patients

☐ Litigation

☐ Data Breaches

☐ Information used for outcome payments

☐ Other

a. **Please Specify:**

____

14. If you were asked to have your patients' health information included in a data linkage study, which has ethics approval, would you agree?

☐ Yes ☐ No ☐ Maybe ☐ No Answer

15. What would enable or persuade you to share?

____

16. Do you currently share data with your PHN?

☐ Yes ☐ No ☐ I don't know

Thank you for your time and feedback

Section D: Providing Access To Data For Research

With access to granularized data (i.e. data that is not aggregated or pooled) it is possible to get a more thorough understanding of the patterns in the data to easily identify areas of interest.

The following questions focus on your comfort level of providing access to the following information for the use in research with appropriate ethics approval which includes stringent collection and management controls.

(Note: All of the following relate to patient information)

	Very comfortable	Somewhat comfortable	Uncertain	Somewhat uncomfortable	Very uncomfortable	No answer
Demographic information (i.e. race, age, gender)	☐	☐	☐	☐	☐	☐
Location information (i.e. post code)	☐	☐	☐	☐	☐	☐
General medical information (i.e. height, weight, blood pressure)	☐	☐	☐	☐	☐	☐
Family medical history (i.e. maternal / paternal health information)	☐	☐	☐	☐	☐	☐
Community care information (i.e. GP visits, blood test results)	☐	☐	☐	☐	☐	☐
Hospital medical information (i.e. outcome of medical procedures)	☐	☐	☐	☐	☐	☐
Personal information (i.e. name, birthdate, ID Number)	☐	☐	☐	☐	☐	☐

Thank you for your time and feedback

Section E: Trust in Data Collection & Storage Organisation

18. When it comes to trust in storing and managing your practice data, how much trust do you place in the following organisations to use patients' medical information responsibly?

	Very high trust	High trust	Moderate trust	Low trust	No trust at all	No answer
Commonwealth Department of Human Services (Providing PIP payments)	☐	☐	☐	☐	☐	☐
Australian Institute of Health and Welfare (AIHW) [Commonwealth]	☐	☐	☐	☐	☐	☐
My Health Record (ADHA) [Commonwealth]	☐	☐	☐	☐	☐	☐
Australian Bureau of Statistics (ABS) [Commonwealth]	☐	☐	☐	☐	☐	☐
State Department of Health	☐	☐	☐	☐	☐	☐
Primary Health Networks (PHN)	☐	☐	☐	☐	☐	☐
Universities	☐	☐	☐	☐	☐	☐
Royal Australian College of General Practice (RACGP)	☐	☐	☐	☐	☐	☐
Software Vendor (i.e. Best Practice, Medical Director)	☐	☐	☐	☐	☐	☐
Extraction Vendor (i.e. PenCS, Polar)	☐	☐	☐	☐	☐	☐
Consumer Health Tracking Application (i.e. Apple, Google, FitBit, Strava, Garmin, etc.)	☐	☐	☐	☐	☐	☐

Thank you for your time and feedback

Section F: Understanding of Organisations Management and Storage of Data

19. When it comes to understanding how data is managed and stored, how familiar are you with the policies and stringency the following organisations have with storing and managing health data?

	Very familiar	Moderately familiar	Somewhat familiar	Slightly familiar	Not at all familiar	No answer
Commonwealth Department of Human Services (Providing PIP payments)	☐	☐	☐	☐	☐	☐
Australian Institute of Health and Welfare (AIHW) [Commonwealth]	☐	☐	☐	☐	☐	☐
My Health Record (ADHA) [Commonwealth]	☐	☐	☐	☐	☐	☐
Australian Bureau of Statistics (ABS) [Commonwealth]	☐	☐	☐	☐	☐	☐
State Department of Health	☐	☐	☐	☐	☐	☐
Primary Health Networks (PHN)	☐	☐	☐	☐	☐	☐
Universities	☐	☐	☐	☐	☐	☐
Royal Australian College of General Practice (RACGP)	☐	☐	☐	☐	☐	☐
Software Vendor (i.e. Best Practice, Medical Director)	☐	☐	☐	☐	☐	☐
Extraction Vendor (i.e. PenCS, Polar)	☐	☐	☐	☐	☐	☐
Consumer Health Tracking Application (i.e. Apple, Google, FitBit, Strava, Garmin, etc.)	☐	☐	☐	☐	☐	☐

Thank you for your time and feedback

Section G: Concerns Related to Sharing Data for Research

20. What concerns, if any, would prevent you from sharing data with the Commonwealth?

	Very concerning	Somewhat concerning	Uncertain	Somewhat not concerning	Not concerning	No answer
Being adversely compared against other practices	☐	☐	☐	☐	☐	☐
Being adversely compared against other practitioners	☐	☐	☐	☐	☐	☐
Potential loss of patients (e.g. patients not wanting to attend a practice that shares data)	☐	☐	☐	☐	☐	☐
Litigation	☐	☐	☐	☐	☐	☐
Data breaches	☐	☐	☐	☐	☐	☐
Information used for outcome payments	☐	☐	☐	☐	☐	☐

21. How confident are you with your level of understanding pertaining to your obligations under Australia's Privacy Legislation?

- ☐ Extremely confident (I have read the legislation and understand my obligations)
- ☐ Fairly confident (I rely on guidance from RACGP)
- ☐ Somewhat confident (Although I get guidance from RACGP, I am still uncertain as to my obligations)
- ☐ Slightly confident (I haven't had time to become familiar with my obligations, but I know I should)
- ☐ Not confident at all (I don't really know where to get information about my obligations)

Thank you for your time and feedback
